# Anemia, transfusions and hospital outcomes among critically ill patients on prolonged acute mechanical ventilation: a retrospective cohort study

**DOI:** 10.1186/cc6885

**Published:** 2008-04-28

**Authors:** Marya D Zilberberg, Lee S Stern, Daniel P Wiederkehr, John J Doyle, Andrew F Shorr

**Affiliations:** 1 School of Public Health and Health Sciences, University of Massachusetts, North Pleasant Street, Amherst, Massachusetts 01003, USA; 2 Analytica International, Park Avenue South, New York, New York 10016, USA; 3 Division of Pulmonary and Critical Care Medicine, Washington Hospital Center, Irving Street Northwest, Washington, District of Columbia 20010, USA

## Abstract

**Introduction:**

Patients requiring prolonged acute mechanical ventilation (PAMV) represent one-third of those who need mechanical ventilation, but they utilize two-thirds of hospital resources devoted to mechanical ventilation. Measures are needed to optimize the efficiency of care in this population. Both duration of intensive care unit stay and mechanical ventilation are associated with anemia and increased rates of packed red blood cell (pRBC) transfusion. We hypothesized that transfusions among patients receiving PAMV are common and associated with worsened clinical and economic outcomes.

**Methods:**

A retrospective analysis of a large integrated claims database covering a 5-year period (January 2000 to December 2005) was conducted in adult patients receiving PAMV (mechanical ventilation for ≥ 96 hours). The incidence of pRBC transfusions was examined as the main exposure variable, and hospital mortality served as the primary outome, with hospital length of stay and costs being secondary outcomes.

**Results:**

The study cohort included 4,344 hospitalized patients receiving PAMV (55% male, mean age 61.5 ± 16.4 years). Although hemoglobin level upon admission was above 10 g/dl in 75% of patients, 67% (n = 2,912) received at least one transfusion, with a mean of 9.1 ± 12.0 units of pRBCs transfused per patient over the course of hospitalization. In regression models adjusting for confounders, exposure to pRBCs was associated with a 21% increase in the risk for hospital death (95% confidence interval [CI] = 1.00 to 1.48), and marginal increases in length of stay (6.3 days, 95% CI = 5.1 to 7.6) and cost ($48,972, 95% CI = $45,581 to $52,478).

**Conclusion:**

Patients receiving PAMV are at high likelihood of being transfused with multiple units of blood at relatively high hemoglobin levels. Transfusions independently contribute to increased risk for hospital death, length of stay, and costs. Reducing exposure of PAMV patients to blood may represent an attractive target for efforts to improve quality and efficiency of health care delivery in this population.

## Introduction

Patients requiring prolonged acute mechanical ventilation (PAMV), defined as 96 hours of mechanical ventilation (MV) or longer, are a group with high hospital utilization intensity [1]. Although constituting roughly one-third of all hospitalized MV patients, they account for about two-thirds of all the hospital resources allocated to the MV group [1]. For example, in the USA in 2003, PAMV patients occupied 6,728,819 hospital days, at an aggregate annualized hospital cost of over $16 billion [1]. At the same time their hospital mortality of 35% is similar to that observed among ventilated patients who require fewer than 96 hours of MV. Based on age-adjusted and disease-specific incidence rates, this population is projected to more than double by the year 2020, thus mandating increased emphasis on efficiency of health care delivery to patients requiring PAMV [2].

Anemia is a frequent complication of critical illness, and its etiology is multifactorial [3-5]. Despite evidence from a large randomized controlled trial suggesting that tolerating a lower hemoglobin among critically ill patients results in unimpaired outcomes, more recent observational data indicate that adherence to this recommendation is poor across the board, and is worst among patients requiring MV [3,5,6]. At the same time, a large body of work specifically addressing the critically ill points to a strong association of exposure to allogeneic blood with such complications as acute lung injury (ALI), ventilator-associated pneumonia, and blood stream infection (BSI) [7-11], and morbidity and attendant hospital resource utilization stemming from such complications may be avoided by more restrictive use of allogeneic blood [12-15].

Because by virtue of having a prolonged critical illness the PAMV population is at greater risk for exposure to packed red blood cell (pRBC) transfusions [16], we hypothesized that in this population more liberal use of allogeneic blood is associated with worse clinical and economic outcomes. Conversely, it would follow that a more restrictive approach to transfusions might result in fewer complications, better outcomes, and thus better quality health care and more efficient health care delivery.

## Materials and methods

### Human subjects protection

Approval was obtained from the Institutional Review Board of the Henry Ford Health System. No informed consent was required because the study involved the use of de-identified claims data.

### Data source

We performed a retrospective cohort study within the Henry Ford Health System (HFHS) database. HFHS is a large, vertically integrated health care system that includes seven hospitals serving the primary and specialty health care needs of residents in the Midwestern USA. The care provided includes more than 2.5 million patient contacts, 20,000 ambulatory surgeries, and 65,000 hospital admissions annually. Most of the care is provided under system-affiliated, salaried physician groups with nearly 900 physicians in more than 40 specialties. Approximately 60% of HFHS members are enrolled in a large nonprofit, mixed-model health maintenance organization. This subset population includes a substantial number of both Medicare (n = 16,000) and Medicaid (n = 22,000) enrollees.

### Cohort identification

In the present analysis we used data from all hospital admissions that took place between January 2000 and December 2005. Patients were included if they were 18 years old or older and had charges associated with at least one procedure code for insertion of an endotracheal tube for MV and at least one code for 96 continuous hours of ventilation. The index date was defined as the date of the first hospital admission containing these codes. Patients on dialysis before the index admission and with a diagnosis code for chronic renal failure were not included in the analysis. Additional file 1 presents the inclusion criteria and associated procedure codes for study patients.

### Exposure variables

The primary exposure of interest was transfusion of pRBCs during the entire period of hospitalization, based on blood bank data linked to specific patient encounters. A transfusion episode was identified by the receipt of at least one unit of pRBCs on any given day of hospitalization. An additional measure of transfusion exposure was units of RBCs transfused per patient. Transfusion exposure was further stratified based on the baseline, pretransfusion, and nadir hemoglobin. Baseline hemoglobin was calculated using the first laboratory measurement on the date of admission; or, if this was not available, the last measurement on the day before admission; or, if none on that day, the first laboratory measurement on the day after admission. Pretransfusion hemoglobin was defined as the lowest hemoglobin measured on or the day before the day of transfusion. Pretransfusion hemoglobin was divided into the following categories: <7 g/dl, 7 to 8 g/dl, 8 to 9 g/dl, 9 to 10 g/dl, and ≥ 10 g/dl [3]. Nadir hemoglobin was defined as the lowest hemoglobin recorded for a patient during the inpatient stay. PAMV patients who received transfused blood were compared with those who did not, based on demographic and clinical characteristics. The burden of chronic illness was assessed using the modified Charlson Comorbidity Index score [17]. Using International Classification of Diseases, ninth revision (ICD-9) codes, 16 weighted co-morbidity variables were defined: a patient's total score (the sum of the scores from each condition present; maximum 34) indicated his or her chronic disease burden.

### Outcome measures

The primary outcome measure was hospital mortality, compared between transfused and nontransfused PAMV patients. Resource utilization (hospital length of stay), hospital costs, discharge hemoglobin, and destination served as the secondary outcomes of interest. Although discharge hemoglobin and destination were examined in a descriptive manner only, hospital mortality, length of stay (LOS) and costs were looked at in multivariable models (see Statistical analyses, below).

### Statistical analyses

Values are expressed as percentages and mean ± standard deviation, and P < 0.05 was considered to represent statistical significance.

Frequencies (for categorical variables) and distributions (for continuous variables), from the index admission date to the date of hospital discharge, were compared using χ^2 ^and Student's *t*-tests, respectively, for all normal data stratified by transfused versus nontransfused patients. Mann-Whitney tests were applied to hospital LOS and cost to assess the significance of differences across exposure to pRBCs. The independent contribution of transfusion exposure to hospital mortality, LOS, and costs was assessed in multivariable models. Although hospital mortality was examined in a logistic regression, the attributable hospital LOS and costs were computed in linear regressions. Because both LOS and costs have highly skewed distributions, the values were log-transformed for the models, and the resultant coefficients were retransformed using the Duan smearing method [18] to yield incremental days and dollars, respectively. The co-variates initially included in the multivariate analyses were patient demographics (age, sex, and race), comorbidities (Charlson Comorbidity Index score), and baseline and nadir hemoglobin levels. Additional variables examined as potential confounders of the relationship between transfusion exposure and outcomes were such complications as hospital-acquired pneumonia (HAP) and BSI (based on the presence of an ICD-9-CM code for pneumonia, bacteremia, or septicemia; see Additional file 1). Finally, potential confounding by such markers of blood loss as gastrointestinal endoscopy, or such surgical procedures as abdominal, cardiac (on-pump and off-pump), and orthopedic surgeries was examined. Those covariates with *P *> 0.2 were eliminated in a manual backward selection process. Diagnostics were performed to ensure the validity of each final model.

## Results

### Cohort characteristics

We identified 4,344 hospitalized patients with PAMV during the time frame examined. Most members of the study population were male (54.5%) and African-American (57.6%), with the mean age being 61.5 ± 16.4 years and the mean Charlson Comorbidity Index score being 7.3 ± 3.6.

The transfusion rate in the cohort was 67.0% (n = 2,912). Transfused patients were older (62.0 ± 16.5 years versus 60.4 ± 16.2 years; *P *= 0.0014), more likely to be female (46.8% versus 42.9%; *P *= 0.018), had a significantly higher chronic disease burden (Charlson Comorbidity Index scores of 7.8 ± 3.6 versus 6.2 ± 3.3; *P *< 0.0001), a lower mean baseline hemoglobin (11.1 ± 2.4 g/dl versus 13.0 ± 2.0 g/dl; *P *< 0.0001), and a lower mean nadir hemoglobin (7.3 ± 1.1 g/dl versus 9.9 ± 1.7 g/dl; *P *< 0.0001) than did those who did not require a transfusion (Table 1). The distribution of the top 5 admitting diagnoses is shown in Table 2. Acute respiratory failure was the most frequent admitting diagnosis in both patient groups, namely those who required a transfusion and those who did not (5.1% versus 12.4%).

**Table 1:** Baseline demographic and clinical characteristics of patients on PAMV by transfusion status

Characteristic	Transfused (n = 2,912)	Nontransfused (n = 1,432)	*P *value
Sex (% female)	46.8	42.9	0.018
Age (years; mean ± SD)	62.0 ± 16.5	60.4 ± 16.2	0.0014
Race (%)			
African American	55.5	61.9	<0.0001
Caucasian	36.9	32.7	
Other	8.6	5.4	
Chronic concomitant comorbidities, %			
Cardiac disease	88.0	79.8	<0.0001
Hypertension	69.4	65.9	0.02
Anemia	64.7	22.8	<0.0001
Pulmonary disease	64.6	65.9	0.43
Gastrointestinal disease	57.6	31.1	<0.0001
Central nervous system disease	50.8	44.8	0.0002
Cancer	39.1	26.1	<0.0001
Immunologic disease	38.9	22.8	<0.0001
Thromboembolic disease	38.5	30.0	<0.0001
Renal disease	35.3	15.8	<0.0001
Diabetes	31.6	26.1	0.0002
Musculoskeletal disease	31.4	14.6	<0.0001
Peripheral vascular disease	29.2	26.9	<0.0001
Cerebrovascular disease	28.7	21.7	0.11
Primary hematologic disease	15.2	7.5	<0.0001

PAMV, prolonged acute mechanical ventilation; SD, standard deviation.

**Table 2:** Distribution of top 5 primary diagnoses among patients on PAMV by transfusion status

Admitting diagnoses (%)	Transfused (n = 2,912)	Nontransfused (n = 1,432)
Acute respiratory failure	5.1	12.4
Heart failure	4.1	6.1
Subendocardial AMI	4.0	3.4
Pneumonia	3.7	5.0
Intracerebral hemorrhage	2.3	3.6

AMI, acute myocardial infarction; PAMV, prolonged acute mechanical ventilation.

### Hemoglobin and transfusions

**Figure 1:**
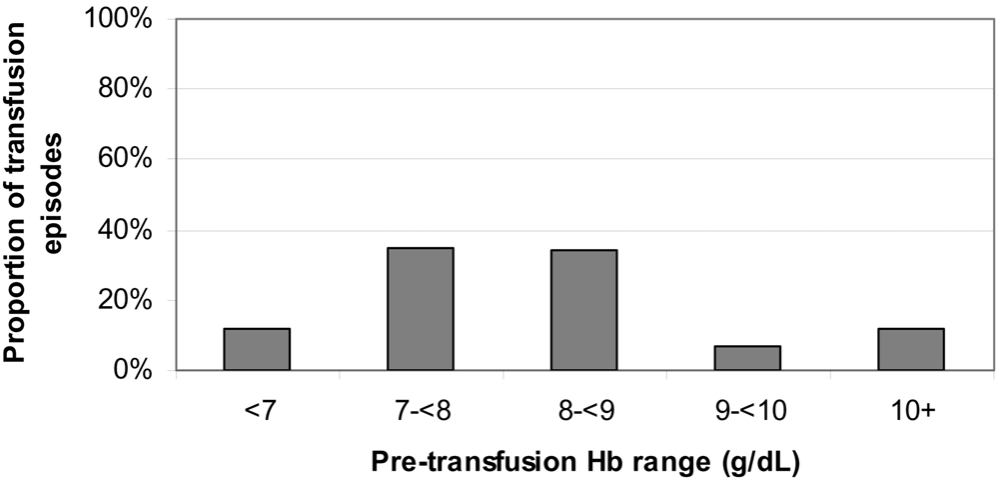
Transfusion episodes occurring at each level of pretransfusion hemoglobin among transfused patients on PAMV. Hb, hemoglobin; PAMV, prolonged acute mechanical ventilation.

The total number of transfusion episodes during the index admission was 7,787 among 2,912 transfused patients. Each patient who was transfused received an average of 3.2 ± 2.8 units/transfusion episode and a total 9.1 ± 12.0 units of blood over the course of the hospitalization. Mean pretransfusion hemoglobin for the 7,787 transfusion episodes was 8.2 ± 1.4 g/dl. The majority of transfusion episodes (69% total) occurred at pretransfusion hemoglobin levels 7 to <8 g/dl (35%; mean units of blood/transfusion episode 3.1 ± 2.9) and 8 to <9 g/dl (34%; mean units of blood/transfusion episode 3.0 ± 3.6; Figure 1). Only 12% of all transfusion episodes in the cohort occurred at the pretransfusion hemoglobin below 7 g/dl, and in this group the mean number of units/transfusion episode was high (5.3 ± 6.5). Conversely, 7% of the episodes occurred in the setting of a pretransfusion hemoglobin above 10 g/dl.

### Outcomes

Crude hospital mortality was significantly higher among PAMV patients who underwent transfusion as compared with those who did not (odds ratio [OR] = 1.51, 95% confidence interval [CI] = 1.31 to 1.75; *P *< 0.0001). Compared with nontransfused patients, transfused patients were also more likely to experience HAP (OR = 1.63, 95% CI = 1.38 to 1.93) or BSI (OR = 2.90, 95% CI = 2.53 to 3.34; *P *< 0.0001 for each) and to have undergone a procedure serving as a marker of high bleeding risk (*P *< 0.0001 for all five procedures). Similarly, transfused patients had a significantly greater hospital LOS and corresponding total hospital costs than did nontransfused patients (Table 3). Of the patients discharged alive, the odds of being discharged directly home were significantly lower among those transfused (OR = 0.38, 95% CI = 0.32 to 0.44; *P *< 0.0001) than those who were not transfused. Conversely, a discharge to an intermediate health care facility was 2.64 (95% CI = 2.25 to 3.11; *P *< 0.0001) times as likely in the transfused group. Finally, despite a high transfusion rate, those transfused were discharged with significantly lower hemoglobin than those who did not receive any transfusions (9.8 ± 1.4 g/dl versus 10.8 ± 1.8 g/dl; *P *< 0.0001; Table 3).

**Table 3:** Hospital events and unadjusted outcomes among patients on PAMV by transfusion status

Events and outcomes	Transfused (n = 2,912)	Nontransfused (n = 1,432)	*P *value
Complications (%)			
Pneumonia	86.2	79.3	<0.0001
Blood stream infection	49.7	20.1	<0.0001
Tracheostomy	71.9	69.8	0.16
Processes of care			
Gastrointestinal endoscopy	31.2	12.4	<0.0001
Abdominal surgery	47	18.4	<0.0001
Cardiac surgery (on-pump)	9.2	0.7	<0.0001
Cardiac surgery (off-pump)	9.9	2.7	<0.0001
Orthopedic surgery	9.3	1.8	<0.0001
Hospital mortality (%)	32.2	23.9	<0.0001
Discharge destination (n [%])			
Home	433 (22.0)	465 (42.7)	<0.0001
Skilled nursing facility	496 (25.1)	200 (18.4)	
Rehabilitation center	382 (19.4)	143 (13.1)	
Home health service	275 (13.9)	147 (13.5)	
Rehabilitation with long-term ventilator care	150 (7.6)	39 (3.6)	
Hospice	52 (2.6)	20 (1.8)	
Other health facility	151 (7.7)	47 (4.3)	
Other/unknown	34 (1.7)	29 (2.7)	
Hemoglobin (mean ± SD)			
Baseline	11.1 ± 2.4	13.0 ± 2.0	<0.0001
Nadir	7.3 ± 1.1	9.9 ± 1.7	<0.0001
Discharge	9.8 ± 1.4	10.8 ± 1.8	<0.0001
LOS			
Mean ± SD	29.6 ± 22.5	15.5 ± 10.9	<0.0001
25th percentile	15	9	
50th percentile	24	13	
75th percentile	37	19	
Charges			
Mean ± SD	101,828 ± 72,925	239,312 ± 187,882	<0.0001
25th percentile	55,294	115,870	
50th percentile	83,537	185,542	
75th percentile	126,623	294,389	

LOS, length of stay; PAMV, prolonged acute mechanical ventilation; SD, standard deviation.

Regression modeling confirmed the independent contribution of pRBC transfusions to hospital mortality, LOS, and aggregate costs of hospitalization. Thus, in a logistic regression, exposure to allogeneic blood was associated with a 21% (95% CI = 1.00 to 1.48) increase in the risk of death, and linear models suggested that transfusions alone were responsible for a marginal 6.3 (95% CI = 5.12 to 7.62) day increase in hospital LOS and $48,972 (95% CI = $45,582 to $52,478) increase in hospital costs (Table 4).

**Table 4:** Mortality, hospital LOS, and costs attributable to transfusion exposure among patients on PAMV

Variable	Adjusted estimate	95% confidence interval
Hospital mortality (adjusted odds ratio)	1.21	1.00 to 1.48
Hospital LOS (days)	6.33	5.12 to 7.62
Hospital costs ($)	$48,973	$45,582 to $52,478

Each estimate adjusted for the following confounding variables: age, sex, race, Charlson Comorbidity Index, baseline and nadir hemoglobin, hospital-acquired pneumonia, blood stream infection, gastrointestinal endoscopy, abdominal surgery, cardiac surgery (on and off bypass), and orthopedic surgery. Mortality outcomes were adjusted additionally for hospital LOS. Hospital LOS and cost outcomes were adjusted for mortality. LOS, length of stay; PAMV, prolonged acute mechanical ventilation.

## Discussion

In the present study we have demonstrated that transfusions are commonly used among patients requiring PAMV, occur at relatively liberal hemoglobin triggers, and are associated with worsened hospital outcomes, including mortality, LOS, and costs. We have also shown that PAMV patients undergoing a transfusion are less likely to be discharged home and, despite a high transfusion burden, leave the hospital with a lower hemoglobin level than those not undergoing a transfusion. Specifically, we have documented a 67% transfusion rate among these patients, with the attendant attributable mortality, LOS, and cost increases of 21%, 6.3 days, and $48,972, respectively. Importantly, these numbers reflect the adjustment for potential contributions to these outcomes of such high risk and costly events as nosocomial infections (HAP and BSI) and gastrointestinal bleeding, and cardiac, abdominal and orthopedic surgeries.

Health care costs in the USA have reached an unprecedented $2.1 trillion, representing 16% of the gross domestic product [19]. A large proportion of this price tag, approximately one-third, is attributed to hospital expenses, and these have been rising steadily [20]. The intensive care unit (ICU), in turn, is a high-intensity use area of the hospital, whose utilization by the Medicare population alone has grown by over 12% in a recent 5-year period [21]. MV, whose attributable cost is $1,500/day in 2002 US dollars, is the single greatest driver of the ICU costs [22]. Illustrating this, the 2003 aggregate hospital costs for all patients undergoing any MV were $25 billion, 64% of which (or $16 billion) was consumed by patients requiring PAMV [1]. Furthermore, given the historic approximately 6% crude annual growth in the volume of PAMV in US hospitals, age-specific incidence change in PAMV over time, and the age-adjusted US population growth, PAMV patients are likely to number over 600,000 cases by year 2020, more than doubling their current numbers [2]. Such substantial utilization of health care resources demands closer scrutiny.

Given that hospital survival rate among patients on PAMV is comparable to that among patients requiring shorter term ventilatory support [1], institution of measures that are directed at optimizing efficiency of health care delivery to this population are critical. Allogeneic blood transfusion is one area of health care delivery in which the penetration of evidence-based practices has remained suboptimal [3,5]. Despite the fact that a randomized controlled trial performed a decade ago confirmed safety of reducing hemoglobin transfusion triggers among critically ill from the traditional 10 g/dl to 7 g/dl [23], the mean pretransfusion hemoglobin remains in the region of 8.5 g/dL among all ICU patients, and is even higher among those requiring MV [3,5,6]. Our study demonstrates nonadherence not only to this recommendation but also to the guidance supporting the administration of only 1 unit of pRBCs per transfusion episode, as was done in the TRICC (Transfusion Requirements in Critical Care) trial [23], because we have observed that on average a transfused PAMV patient receives more than 3 units of blood in a single transfusion episode.

Unfortunately, this nonadherence to recommendations based on the best evidence is not without consequences. Evidence points to a strong association between exposure to blood transfusions and such complications as ventilator-associated pneumonia and BSIs [9,10,24], as well as multiple other infectious and immune complications [25-27]. Evidence for the connection between acute respiratory distress syndrome (ARDS) and transfusion exposure is also mounting. For example, not only did the difference in the ARDS incidence nearly reach statistical significance in the TRICC trial (*P *= 0.06), with the more favorable results in the restrictive arm [23], but also several prospective cohort studies have strengthened this association [7,8,11], particularly because some were able to detect a dose-response relationship between the magnitude of blood exposure and risk for subsequent development of ARDS [7,11]. Giving further credence to this causal relationship is a recent report from the Mayo clinic [13], in which adherence to a restrictive transfusion protocol along with the use of a lung-protective ventilation strategy resulted in a reduction in ALI incidence from 28% to 10%. In general, it is estimated that adopting restrictive triggers more ubiquitously could result in avoidance of nearly 40,000 acute complications associated with transfusions, and, more specifically, approximately 17,000 cases of ARDS alone [14,15]. This may even be an under-estimate, given the results of the nested case-control study conducted by Gajic and coworkers [28], in which ALI was prospectively observed in 8% of all patients exposed to blood products. Our study, by focusing on the population of MV patients at greatest risk for transfusions (namely those receiving PAMV), deepens our understanding not only of transfusion practices in PAMV patients but also of how altering these practices in line with the best evidence might result in lower costs and better outcomes for patients. Given that more than a half of all of the observed transfusion episodes were administered at or above a hemoglobin concentration of 8 g/dl, and given the noted contribution of allogeneic pRBC exposure to mortality, hospital LOS, and costs in the PAMV population, there is a substantial opportunity to improve these outcomes via an evidence-driven reduction in transfusion triggers.

Our study has a number of limitations. First, its retrospective nature lends itself to multiple forms of bias, the most important of which is a selection bias. We have mitigated this possibility by employing uniform inclusion criteria that are based on a strict definition of the population, as well as by following all patients to the hard end-points of hospital discharge or death. Second, having been performed in a single health care system, the underlying population characteristics may limit the generalizability of our findings. Third, the accuracy of the identification of hospital complications (HAP and BSI) and such processes of care as the surgical procedures we adjusted for may be at least somewhat questionable, because we utilized administrative coding, and not clinical data, to define these conditions. Although it is possible that the first two are prone to being over-reported in the hospital's billing system, particularly for the sicker patients, the reporting of surgical procedures is less likely to have an inherent bias. Fourth, because of its observational nature, and although we attempted to adjust for confounders, the possibility of residual confounding remains. Finally, our study was performed before widespread utilization of leukoreduction, and thus it may overestimate the association between transfusions and such adverse outcomes as infectious complications or ALI. However, this potential inflation of the association, if present, is likely to be small, because a recent randomized controlled trial failed to observe a reduction in the incidence of either infection or ALI in the group exposed to leukoreduced blood [29,30].

## Conclusion

In summary, we have demonstrated that two-thirds of all patients on PAMV are exposed to allogeneic blood during their hospitalization. Furthermore, the total amount of blood is, on average, a staggering 9 units per transfused patient. Even with a pretransfusion hemoglobin of >7 g/dl, the number of units administered per one episode of transfusion is over 3. This potential overuse of allogeneic blood is associated with a 21% increase in adjusted hospital mortality, and an incremental hospital LOS and costs of 6.3 days and $48,972, respectively. Our data support the idea that evidence-based transfusion practices in this population of critically ill patients may be one of the ways to improve quality and efficiency of health care delivery.

## Key messages

• Patients on PAMV (defined as MV for ≥ 96 hours) are at a high risk for transfusion with pRBCs.

• Only 12% of all transfusion episodes took place at a hemoglobinn concentration below 7 g/dL, with an additional 35% of episodes occurring between 7 and <8 g/dl.

• On average, a transfusion episode consists of 3.2 ± 2.8 units of pRBCs.

• Exposure to allogeneic blood is associated with an adjusted increase in hospital mortality of 21%, hospital LOS of 6.3 days, and hospital costs of $48,972.

## Abbreviations

ALI = acute lung injury; ARDS = acute respiratory distress syndrome; BSI = blood stream infection; CI = confidence interval; HAP = hospital-acquired pneumonia; HFHS = Henry Ford Health System; ICD-9 = International Classification of Diseases, ninth revision; ICU = intensive care unit; LOS = length of stay; MV = mechanical ventilation; OR = odds ratio; PAMV = prolonged acute mechanical ventilation; pRBC = packed red blood cell.

## Competing interests

At the time of this study, MDZ was an employee of Ortho Biotech Clinical Affairs, LLC (Bridgewater, NJ, USA). She currently serves as a consultant to Ortho Biotech Clinical Affairs, LLC, and is a stockholder in Johnson & Johnson (New Brunswick, NJ, USA), its parent company. At the time of this study LSS, DPW, and JJD were employees of Analytica International (New York, NY, USA), which has received research funds from Ortho Biotech Clinical Affairs, LLC. AFS is a consultant to and has received funding from Ortho Biotech Clinical Affairs, LLC.

## Authors' contributions

MDZ, DPW, and AFS were responsible for study design, data interpretation, and drafting the manuscript. LSS and JJD were responsible for study design, data analyses, and data interpretation. All authors read and approved the final manuscript.

## Supplementary Material

Additional file 1 Inclusion criteria and associated procedure codes for study patients. Presented are the inclusion criteria and associated procedure codes for study patients. Click here for file
