# Supplementary material for: Anemia, transfusions and hospital outcomes among critically ill patients on prolonged acute mechanical ventilation: a retrospective cohort study
Source: Crit Care. 2008 Apr 28;12(2):R60. doi: 10.1186/cc6885 (PMC2447616; doi:10.1186/cc6885)
Supplement: Additional file 1 — Inclusion criteria and associated procedure codes for study patients. Presented are the inclusion criteria and associated procedure codes for study patients. [file cc6885-S1.doc]

**Additional file 1**

| **Inclusion Criteria** | **Codes** |
| --- | --- |
| >18 years of age |  |
| ≥1 one procedure code within 1 hospital admission from January 1, 2000 to December 31, 2005 for insertion of a mechanical ventilator | ICD-9 96.01, 96.02, 96.04, 96.05, 31.1, 31.21, 31.29 or CPT 94656 |
| 96 hours of continuous mechanical ventilation | ICD-9 96.72 |
| NOT on dialysis prior to index admission with a diagnosis code for chronic renal failure | CPT 90920, 90921, 90924, 90925, 90937, 90947, 99512, 99559 or ICD-9 39.95, 54.98 and ICD-9 585, 403.01, 403.91. 404.92, 404.93, 285.21 |
| Blood stream infections (BSI) | 001.xx-005.xx, 008.xx-018.xx, 020.x-027.x, 030.xx-041.xx, 090.xx-098.xx, 100.x-104.x, 110.xx-118.xx, 320.xx, 322.x, 324.x, 325, 420.xx, 421.x, 451.xx, 461.xx- 465.xx, 481, 482.xx, 485, 486, 491.21, 494.x, 510.x, 513.x, 540.x, 541, 542, 562.01, 562.03, 562.11, 562.13, 566, 567.x, 569.5, 569.83, 572.0, 572.1, 575.0, 590.xx, 597.xx, 599.0, 601.x, 614.x, 615.x, 616.xx, 681.xx, 682.x, 683, 686.xx, 711.0x, 730.xx, 790.7, 996.6x, 998.5x, 999.3  **AND one of the following:**  785.5x, 458.x, 348.3, 293.xx, 348.1, 287.4, 287.5, 286.9, 286.6, 570, 573.4, 584.x |
| Hospital acquired pneumonia (HAP) | 010-012.x, 0.18.xx, 020.3-020.5, 021.2, 022.1, 031.0, 032.1-032.3, 033.x, 034.0, 039.1, 052.1, 055.1, 073.0, 095.1, 114.0, 114.4, 114.5, 115.05, 115.15, 115.95, 130.4, 136.3, 137.0, 460-466.xx, 480-487.x, 511.1, 513.0 |

| **Event** | **CPT Codes for Inpatient Events Defined as:** |
| --- | --- |
| Gastrointestinal Endoscopy | 43200-43272, 44360-44397, 45300-45387 |
| Abdominal Surgery | 43020, 43045-43135, 43300-43425, 43460, 43496, 43499 43500-43641, 43750, 43800-44160, 44300-44346, 44602-44900, 44950-44960, 45110-45170, 45500, 45505, 45540-45825, 45999, 47010, 47015, 47100-47362, 47380-1, 47399, 47400-47480, 47600-47999, 48000-48100, 48120-48180, 48500-48510, 48520-48999, 49000-49010, 49020, 49040, 49060, 49062-49255, 49505-49611, 49904-49999 |
| Cardiac Surgery | 33015-33250, 33251*, 33253, 33261*, 33300, 33305*, 33310, 33315*, 33320-33321, 33322*, 33330-33332, 33335*, 33400*, 33401, 33403*, 33404, 33405-33410*, 33411-33417, 33422*, 33425-33427*, 33430*, 33460*, 33463-33464, 33465*, 33468, 33472, 33474*, 33475-33478, 33496*, 33500*, 33501-33503, 33504*, 33505-33506, 33510-33732, 33736*, 33737-33813, 33814*, 33820-33852, 33853*, 33860-33870*, 33875(*), 33877(*), 33910*, 33915, 33916*, 33917-33918, 33919*, 33920, 33922*, 33924-33980, 33641*, 33702*, 33710*, 33720*, 33999 |
| Orthopedic Surgery | 20150-20205, 20220-20251, 20520-20525, 20650, 20670-20694, 20802-20808, 20838, 20900-20938, 20950-20969, 22100-22226, 22318-22328, 22548-22899, 23000-23020, 23035-23332, 23395-23491, 23515, 23530-23532, 23550-23552, 23585, 23615-23616, 23630, 23660, 23670, 23680, 23800-23929, 23935-24201, 24301-24498, 24515-24516, 24545-24546, 24575, 24579, 24586-24587, 24615, 24635, 24665-24666, 24685-24940, 25000-25025, 25035-25251, 25260-25492, 25515, 25525-25526, 25545, 25574-25575, 25620, 25628, 25645, 25652, 25670, 25676, 25685, 25695-25999, 26992-27091, 27097-27187, 27202-27215, 27217-27218, 27226-27228, 27236, 27244-27245, 27248, 27253-27259, 27280-27299, 27303-27365, 27372-27499, 27506-27507, 27511-27514, 27519, 27524, 27535-27536, 27540, 27556-27558, 27566, 27580-27599, 27600-27602, 27605-27647, 27650-27745, 27758-27759, 27766, 27784, 27792, 27814, 27822-27823, 27826-27829, 27832, 27846-27848, 27870-27899 |

*Indicates procedure with cardiopulmonary bypass (on-pump)

| **Event** | **ICD-9 Codes for Inpatient Events Defined as:** |
| --- | --- |
| Gastrointestinal Endoscopy | 42.22-42.24, 44.12-44.14, 45.12-45.14, 45.16, 45.22-45.25, 42.33, 43.41, 44.22, 44.43, 45.30, 45.42, 45.43, 48.22-48.24, 48.36 |
| Abdominal Surgery | 42.0x, 42.1x, 42.21, 42.31, 42.32, 42.39, 42.4x, 42.5x, 42.6x, 42.7, 42.8x, 42.91, 43.0, 43.1x, 43.3, 43.42, 43.49, 43.5, 43.6, 43.7, 43.8x, 43.9x, 44.0x, 44.11, 44.15, 44.21, 44.29, 44.3x, 44.40-44.42, 44.44, 44.49, 44.5, 44.6x, 44.91, 44.99x, 45.0x, 45.11, 45.15, 45.21, 45.26, 45.31-45.34, 45.41-45.49, 45.5x, 45.6x, 45.7x, 45.8, 45.9x, 46.0x, 46.1x, 46.2x, 46.3x, 46.4x, 46.5x, 46.6x, 46.7x, 46.80-46.82, 46.91-46.94, 46.97, 46.99,-, 47.09, 47.19, 47.2, 47.9x, 48.0, 48.1, 48.21, 48.25, 48.4x, 48.5, 48.6x, 48.7x, 50.0, 50.12, 50.19, 50.2x, 50.3, 50.4, 50.5x, 50.6x, 51.02-51.04, 51.13, 51.19, 51.21, 51.22, 51.3x, 51.4x, 51.5x, 51.61-51.63, 51.69, 51.7x, 51.81-51.83, 51.89, 51.91-51.95, 51.99, 52.0x, 52.12, 52.19, 52.22, 52.3, 52.4, 52.5x, 52.6, 52.7, 52.8x, 52.92, 52.95, 52.96, 52.99, 53.0x, 53.1x, 53.2x, 53.3x, 53.4x, 53.5x, 53.6x, 53.7, 53.8x, 53.9, 54.0, 54.1x, 54.22, 54.23, 54.29, 54.3, 54.4, 54.59, 54.6x, 54.7x, 54.92-54.95 |
| Cardiac Surgery* | 35.1x, 35.2x, 35.3x, 35.42, 35.50, 35.51, 35.53, 35.54, 35.6x, 35.7x, 35.8x, 35.91-35.95, 35.98, 35.99, 36.03, 36.1x, 36.2, 36.31, 36.39, 36.9x, 37.1x, 37.24, 37.25, 37.29, 37.3x, 37.4, 37.5, 37.6x, 37.74-37.77, 37.80, 37.85-37.89, 37.91, 37.94-37.99 |
| Orthopedic Surgery | 77.00-77.03, 77.05-77.07, 77.09, 77.10-77.13, 77.15-77.17, 77.19, 77.20-77.23, 77.25-77.27, 77.29, 77.30-77.33, 77.35-77.37, 77.39, 77.40-77.43, 77.45-77.47, 77.49, 77.60-77.63, 77.65-77.67, 77.69, 77.70-77.73, 77.75-77.77, 77.79, 77.80-77.83, 77.85-77.87, 77.89, 77.90-77.93, 77.95-77.97, 77.99, 78.x0, 78.x1, 78.x2, 78.x3, 78.x5, 78.x6, 78.x7, 78.x9, 79.20-79.23, 79.25-79.27, 79.29, 79.30-79.33, 79.35-79.37, 79.39, 79.50-79.53, 79.55-79.57, 79.59, 79.60-79.63, 79.65-79.67, 79.69, 79.80-79.83, 79.85-79.87, 79.89, 79.9, 80.00-80.03, 80.05-80.07, 80.09, 80.10-80.13, 80.15-80.17, 80.19, 80.40-80.43, 80.45-80.47, 80.49, 80.50, 80.51, 80.59, 80.6, 80.70-80.73, 80.75-80.77, 80.79, 80.80-80.83, 80.85-80.87, 80.89, 80.90-80.93, 80.95-80.97, 80.99, 81.0x, 81.11, 81.20-81.25, 81.29, 81.3x, 81.4x, 81.51-81.56, 81.59, 81.61, 81.73, 81.8x, 81.93-81.99, 83.0x, 83.1x, 83.2x, 83.3x, 83.4x, 83.5, 83.6x, 83.7x, 83.8-83.83, 83.85-83.89, 83.91-83.93, 83.99, 84.00, 84.04-84.09, 84.10, 84.13-84.19, 84.23, 84.24, 84.27-84.29, 84.3, 84.40, 84.44, 84.48, 84.91-84.99 |

*Additional presence of code 39.61 indicates procedure with cardiopulmonary bypass (on-pump)
